# Investigation of the Incorporation of Cerium Ions in MCVD-Silica Glass Preforms for Remote Optical Fiber Radiation Dosimetry

**DOI:** 10.3390/s21103362

**Published:** 2021-05-12

**Authors:** Monika Cieslikiewicz-Bouet, Hicham El Hamzaoui, Youcef Ouerdane, Rachid Mahiou, Geneviève Chadeyron, Laurent Bigot, Karen Delplace-Baudelle, Rémi Habert, Stéphane Plus, Andy Cassez, Géraud Bouwmans, Mohamed Bouazaoui, Adriana Morana, Aziz Boukenter, Sylvain Girard, Bruno Capoen

**Affiliations:** 1 Univ-Lille, CNRS, UMR 8523-PhLAM-Physique des Lasers Atomes et Molécules, F-59000 Lille, France; laurent.bigot@univ-lille.fr (L.B.); karen.baudelle@univ-lille.fr (K.D.-B.); remi.habert@univ-lille.fr (R.H.); stephane.plus@univ-lille.fr (S.P.); andy.cassez@univ-lille.fr (A.C.); geraud.bouwmans@univ-lille.fr (G.B.); mohamed.bouazaoui@univ-lille.fr (M.B.); bruno.capoen@univ-lille.fr (B.C.); 2 Laboratoire H. Curien, Univ Lyon, UJM-CNRS-IOGS, 18 Rue du Pr. Benoît Lauras, F-42000 Saint-Etienne, France; ouerdane@univ-st-etienne.fr (Y.O.); adriana.morana@univ-st-etienne.fr (A.M.); aziz.boukenter@univ-st-etienne.fr (A.B.); sylvain.girard@univ-st-etienne.fr (S.G.); 3 Institut de Chimie de Clermont-Ferrand, Université Clermont Auvergne, CNRS, SIGMA Clermont, F-63000 Clermont-Ferrand, France; rachid.mahiou@uca.fr (R.M.); genevieve.chadeyron@sigma-clermont.fr (G.C.)

**Keywords:** cerium, silica glass preforms, photoluminescence, optical fiber sensors, radioluminescence, X-ray dosimetry

## Abstract

The incorporation of Ce^3+^ ions in silicate glasses is a crucial issue for luminescence-based sensing applications. In this article, we report on silica glass preforms doped with cerium ions fabricated by modified chemical vapor deposition (MCVD) under different atmospheres in order to favor the Ce^3+^ oxidation state. Structural analysis and photophysical investigations are performed on the obtained glass rods. The preform fabricated under reducing atmosphere presents the highest photoluminescence (PL) quantum yield (QY). This preform drawn into a 125 µm-optical fiber, with a Ce-doped core diameter of about 40 µm, is characterized to confirm the presence of Ce^3+^ ions inside this optical fiber core. The fiber is then tested in an all-fibered X-ray dosimeter configuration. We demonstrate that this fiber allows the remote monitoring of the X-ray dose rate (flux) through a radioluminescence (RL) signal generated around 460 nm. The response dependence of RL versus dose rate exhibits a linear behavior over five decades, at least from 330 µGy(SiO_2_)/s up to 22.6 Gy(SiO_2_)/s. These results attest the potentialities of the MCVD-made Ce-doped material, obtained under reducing atmosphere, for real-time remote ionizing radiation dosimetry.

## 1. Introduction

Ionizing radiation, since its discovery in 1895 by Wilhelm Röntgen and following research carried out by Henri Becquerel, Pierre and Marie Curie [1], finds a plethora of applications in medicine (diagnostic and treatment), nuclear power supply, fundamental research, industrial manufacturing, sterilization, non-destructive testing, food processing, etc. The usefulness of ionizing radiation must be balanced with its hazards for living organisms and for the environment [2]. Therefore, accurate and precise measurement of the radiation dose is necessary, as well as its sensitivity and repeatability. In the scope of ionizing radiation dosimetry, optical fiber sensors attract a huge interest due to their small size, intrinsic immunity to electromagnetic interferences, flexibility and ability to be remotely interrogated [3,4,5]. They also offer high spatial resolution of the measurement with the possibility to work in hazardous, narrow and constrained environments. Among several radiation dosimetry techniques, the application of optical fibers started with thermoluminescence (TL) [6], and followed by radiation induced attenuation (RIA), optically stimulated luminescence (OSL) and radioluminescence (RL) [7]. For RL based techniques, the scintillation signal from the radiation exposed probe can be recorded, allowing real-time dose-rate measurements, which makes this technique very interesting for applications in medical or severe environmental domains.

Cerium is a rare-earth element with the [Xe] 6s^2^ 4f^1^ 5d^1^ electronic configuration, that exists in different stable valence states (Ce^3+^ and Ce^4+^). These ions have attracted a lot of attention in glass and optical fibers manufacturing, due to their ability to make the glass matrix more resistant against ionizing radiation (notably γ-radiation [8,9,10], X-rays [11]). They also play an important role in the inhibition of photodarkening (PD) in multicomponent glasses [12]. Moreover, only Ce^3+^ ions present optical activity [13], namely a visible luminescence under UV or ionizing radiation. This emission shows fast decay times compatible with time-resolved dosimetry. However, the co-existence of both oxidation states of cerium is observed in glass samples even if a precursor containing only trivalent cerium is used in the glass fabrication. The ratio of cations concentrations [Ce^3+^]/[Ce^4+^] depends on the glass elaboration atmosphere [14], on the presence of silica matrix modifiers or on a reducing agent added to the glass during fabrication [15]. The incorporation of cerium ions into silica glass was investigated by Ishii et al. in the late 1980s [16]. More recently, Cicconi et al. [17] demonstrated the impact of aluminum co-doping in the stabilization of Ce^3+^ ions inside silicate glasses obtained by MCVD method. Engholm et al. [18] have reported on Ce co-doping and improved PD hardening in Yb-based optical fiber lasers. Likewise, Kirchhoff’s group [19] has studied the optical properties of Ce-doped silica multicomponent optical fibers for high power lasers. Furthermore, Ce-doped silica glasses are the subject of investigation for ionizing radiation dosimetry based on the Ce^3+^ ions luminescence properties [20]. For this particular application, the use of pure silica instead of multicomponent glasses presents a great advantage. Indeed, despite the fact that co-doping with Al or P stabilizes the Ce^3+^ ions inside doped silica glasses [17,21], the presence of such elements is suspected to provoke defect formation under ionizing radiations [22,23]. The optical absorption of these defects could degrade the Ce^3+^ scintillation efficiency in the visible domain. Hence, Ce-doped pure silica glasses are considered to be more compatible with dosimetry application. However, up to now, the development of such doped silica glasses was limited to the sol-gel technique [24,25,26,27], yet MCVD remains a reference method to produce optical fiber preforms with a rather smart control of the glass composition. Therefore, we have focused on the investigation of Ce^3+^ incorporation into pure silica matrices by MCVD technique. The application of controlled atmosphere during the fabrication permitted us to favor the Ce(III) ions formation. The structural and optical properties of the elaborated samples were investigated via a set of spectroscopic techniques such as: Raman scattering, optical absorption and photoluminescence. The Ce-doped silica preform with the highest QY was drawn into a 125 µm-diameter optical fiber to achieve a miniaturized radioluminescence-based dosimeter. The RL response of this fiber has been studied in a wide dose-rate range of X-rays.

## 2. Materials and Methods

All studied glass preforms were obtained by conventional MCVD coupled with “solution doping” at FiberTech Lille platform (Lille, France). In our experiments, pure silica tubes (Heraeus F300, Hanau, Germany) were used as substrates. The internal part of tubes was first etched with freon gas (C_2_F_6_) at 1900 °C. Then, pure silica soot was deposited at 1690 °C. This obtained layer was soaked with an ethanol solution of Ce(NO_3_)_3_.6H_2_O at 10 mM concentration for 30 min. After solution removal, the doped silica layer was dried in a He/O_2_ atmosphere on the MCVD lathe and treated thermally with chlorine at 1100 °C to eliminate hydroxyl groups. Then, the porous layer was densified at 1800–1900 °C under a controlled atmosphere and sintered at temperatures ramping from 1980 °C to 2100 °C, prior to undergoing a collapse step at 2200 °C with a traversing burner in the backward direction. To ensure either completely oxidizing, neutral or reducing conditions, we have adapted the process to introduce only O_2_, He or Noxal 3^TM^ mixture (3% of H_2_ in Ar) with the same gas flow in each fabrication. This procedure has been applied during the densification step and the first passes of sintering (in the case of Noxal atmosphere) or during the whole thermal treatment (as for the oxygen and helium atmospheres). Three preforms are hence obtained and are hereafter denoted as P_Oxy_, P_Hel_ and P_Nox_ in reference to the applied atmosphere.

For fiber drawing, the P_Nox_ preform was processed by HF etching to obtain a 5 mm diameter rod. This allowed the direct drawing of the sample, at high temperature (around 2000 °C), into an optical fiber with the targeted dimensions. During the fiber drawing process, low-index polymer coating was applied to ensure optical guiding with a numerical aperture (NA) of about 0.4.

All preforms were cut into thin slices and polished for chemical and optical characterizations. Electron Probe Microanalysis (EPMA) was performed using a Cameca SX 100 microprobe (Cameca, Genevilliers, France).

The Raman spectral analysis was made in a confocal configuration with the aid of a triple-grating T64000 spectrometer (HORIBA JobinYvon, Lille, France) equipped with a liquid nitrogen(LN_2_)-cooled CCD detector and the 514.5 nm Ar^+^ laser line as the excitation source, with a power of 0.2 W. All Raman spectra were recorded at room temperature in the wavenumber range 200–1300 cm^−1^ with a 0.5 cm^−1^ resolution.

Absorption spectra in the UV-VIS spectral domain were recorded at room temperature using a Cary 5000 double-beam spectrophotometer (Agilent, Santa Clara, CA, USA).

Photoluminescence spectra (PL) recordings were made at room temperature using an UV/VIS mini-spectrometer (C10082CA Hamamatsu, Hamamatsu, Japan). For all the samples, excitation has been made perpendicularly to the direction of the signal collection to avoid the re-absorption of the light in the analyzed samples.

The time-resolved luminescence (TRL) experiments were performed with an excitation laser source based on an optical parametric oscillator equipped with a second harmonic generation nonlinear crystal pumped by the third harmonic of a Nd:YAG laser. Characteristics of the selected probe signal are as follows: a pulse width duration 5 ns and a repetition rate 10 Hz. The light (signal) emitted by all samples was spectrally resolved using a grating-based spectrometer with 300 grooves/mm and recorded by a sensitive gated intensified CCD (PI-MAX high speed gated camera, Princeton Instruments, Trenton, NJ, USA) equipped with a time window-delay generator.

The determination of an absolute photoluminescence quantum yield (PL QY) values was performed at room temperature using a C9920-02G PL-QY measurement system (Hamamatsu, Shizuoka, Japan). This system includes both a CCD and a spectrometer for detecting the whole spectral luminescence range.

The fiber transverse cross-section was imaged with a TM1000 tabletop scanning electron microscope (SEM) (Hitachi Ltd., Chiyoda, Japan).

The Ce^3+^ ions distribution over the fiber transverse cross-section, was investigated through micro-photoluminescence (µPL) measurements by using an Aramis (Jobin-Yvon) spectrometer equipped with a CCD camera, a He-Cd ion laser line at 325 nm (energy 3.8 eV), a 2D micro-translation stages and an objective with optical magnification of 40. The microscope spatial resolution was in the order of 4 µm.

The X-ray beam was delivered by the LABHX facility of Laboratoire Hubert Curien (Saint Etienne, France), operating at 100 kV and generating photons of ~40 keV average energy. The X-ray dose rate was driven by the equipment’s electric current. The dose rate is evaluated using an ionization chamber, calibrated to provide the dose rates in Gy(H_2_O)/s. These values were converted in Gy(SiO_2_)/s taking into consideration the ratio of mass attenuation coefficients between water and silica [28]. The maximum dose rate achieved with this experimental setup corresponds to 22.6 Gy (SiO_2_)/s. The RL signals were recorded using single photon avalanche detector associated with a rapid counting module (SR400 from Stanford Research Systems, Sunnyvale, CA, USA).

As for PL measurements, RL spectra were recorded using the same spectrometer (C10082CA, Hamamatsu).

## 3. Results and Discussion

### 3.1. Ce-Doped Preforms

#### 3.1.1. Composition and Structural Properties

Depending on fabrication conditions, three glass preforms were obtained by conventional MCVD combined with “solution doping”. The concentration of Ce element was measured within the three fabricated preforms using Electron Probe Microanalysis (EPMA). The measured cerium concentration varies in the obtained samples, even though the same Ce concentration was applied in the original soaking solution. P_Oxy_, P_Nox_, and P_Hel_ presented a mean Ce concentration of about 220, 430 and 50 atomic ppm respectively. This indicates that both reducing and oxidizing conditions are favorable to cerium incorporation when compared to the inert one. An explanation might be found in the high thermal conductivity of helium, which can lead to an enhanced heat transmission within the soot layer during the high temperature passes. That might provoke the evaporation and removing of Ce volatile specimens. This phenomenon has been already reported for phosphorus-doped SiO_2_ MCVD glasses treated in oxygen and helium gas flows [29].

**Figure 1:**
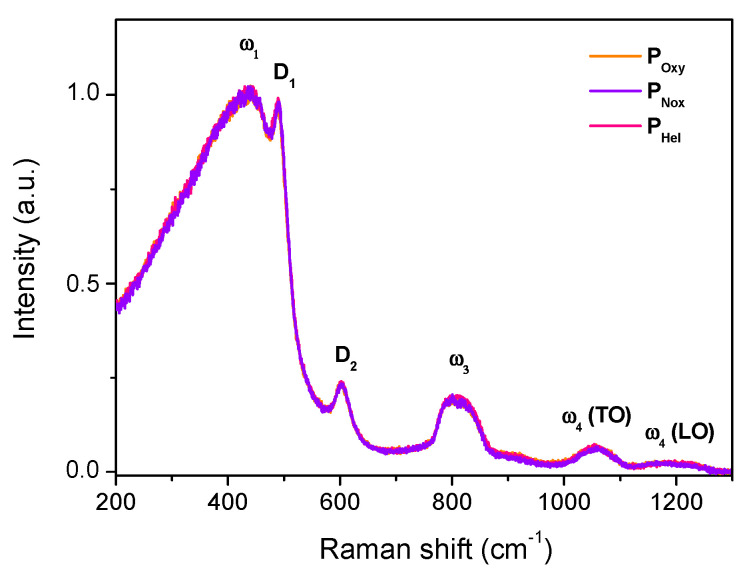
Raman spectra of the three Ce-doped preforms showing the band attribution according to ref. [30].

The microstructure of these three preforms was investigated through Raman spectroscopy. Figure 1 presents the normalized Raman spectra recorded in the core region of Ce-doped MCVD preforms. These spectra show the well-known vitreous SiO_2_ bands [30]. It can be seen that, independently of the atmosphere used during the preform fabrication, the Raman spectra are globally analogous, indicating that there is no significant difference between the glass structure of these three samples. Hence, the elaboration atmosphere does not notably impact the host silica matrix structure.

#### 3.1.2. Optical Absorption

**Figure 2:**
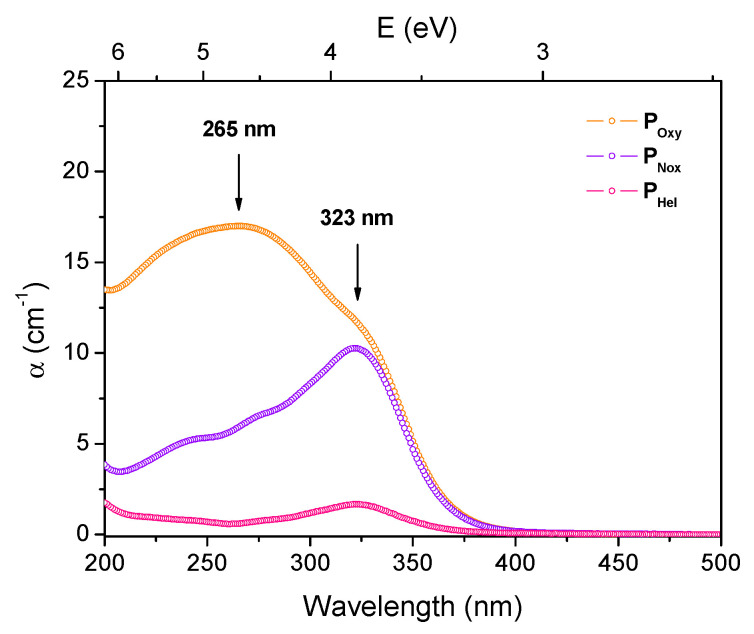
Ce-doped preforms optical absorption spectra.

Figure 2 shows Ce-doped MCVD preforms optical absorption spectra. It can be noticed that the sample prepared under oxidizing conditions exhibits two absorption bands centered around 265 nm and 323 nm. While the first one is assigned to charge transfer transitions from ligands to the quadruply-charged cerium cations (Ce^4+^), the second one states for 4f→5d transition of Ce^3+^ ions [11,31]. However, for the preforms obtained under inert (P_Hel_) or reducing conditions (P_Nox_), the spectra essentially display a main absorption band, peaking around 323 nm, attributed to Ce^3+^ ions. This confirms that, in pure oxygen atmosphere, cerium is stabilized in both tetravalent (Ce^4+^) and trivalent (Ce^3+^) states inside the silica host while the trivalent state (Ce^3+^) is promoted by neutral and reducing atmosphere conditions. Moreover, compared to the inert atmosphere, it can be noted that the Ce^3+^-related absorption band presents higher amplitude when the sample is fabricated under reducing atmosphere. This result agrees with those obtained via EPMA, showing a higher concentration of cerium in P_Nox_ compared to P_Hel_.

The concentration of the two valence species has been estimated from the absorption coefficient measured in Figure 2 and from the absorption cross-sections values reported for Ce^3+^ and Ce^4+^ ions in silica glass [16]. For the P_Oxy_ preform, from the experimental absorption coefficient at 265 nm we estimated the Ce^4+^ concentration ions to be of about 1.7×10^18^ ions/cm^3^. Then, from the mean total Ce concentration, determined by EPMA, we calculated the Ce^3+^ concentration of 3.1 × 10^18^ ions/cm^3^ for P_Oxy_ preform. In the case of P_Nox_ and P_Hel_ samples, considering the absorption coefficients of Ce^3+^ ions at 323 nm and the corresponding absorption cross-section value from [16], the Ce^3+^ ions concentration was estimated to be about 10.2×10^18^ and 1.6×10^18^ ions/cm^3^ for P_Nox_, and P_Hel_, respectively. These values are close to the total concentration of Ce determined using EPMA. Hence, we consider that the concentration of Ce^4+^ ions is negligible in these samples and that cerium is incorporated under its trivalent (Ce^3+^) form.

#### 3.1.3. Photoluminescence Properties

**Figure 3:**
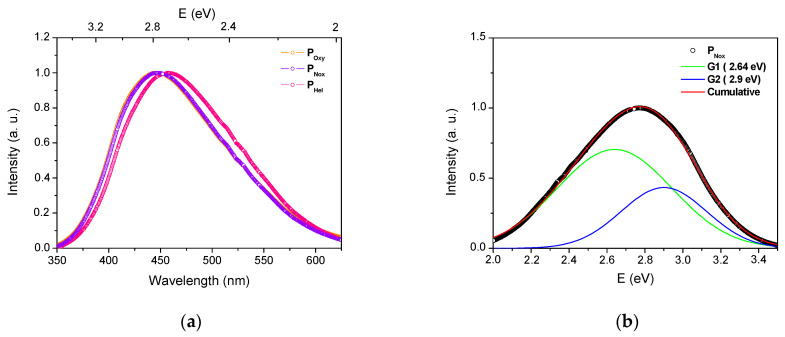
(**a**) Normalized PL spectra of the three Ce-doped preforms under excitation at 320 nm. (**b**) PL spectrum Gaussian shape decomposition for the P_Nox_ sample.

The photoluminescence spectra of the three Ce-doped preforms under excitation at 320 nm are presented in Figure 3a. As expected, all samples show a large visible emission band. However, while both P_Oxy_ and P_Nox_ present the same PL spectrum shape with an emission band peaking around 450 nm, the P_Hel_ PL spectrum exhibits a red shift with a maximum around 460 nm. All samples reveal an asymmetrical PL band, which is attributed to the allowed electric dipole 5d–4f transition of Ce^3+^. The decomposition into two Gaussian functions (G1 and G2) can be made, as shown in the example of P_Nox_ (Figure 3b). Table 1 summarizes the characteristics of the G1 and G2 bands for the three samples. Independently of the fabrication conditions, G1 and G2 bands are located at 2.64 and 2.9 eV, respectively. These two values originate from the spin-orbit splitting of the ground state into sublevels ^2^F_5/2_ and ^2^F_7/2_. The energy difference between the two sublevels was found around 0.26 eV (2097 cm^−1^), in agreement with the typical values reported in the case of Ce^3+^ ions in oxide glasses [32,33].

In Table 1, the contribution of G1 (5d → 4f (^2^F_7/2_)) and G2 (5d → 4f (^2^F_5/2_)) sub-bands to the overall emission band is similar for both P_Oxy_ and P_Nox_. However, in the P_Hel_ case, a higher contribution of the G1 sub-band to the overall emission band is highlighted. The red shift observed in the P_Hel_ case, compared to the P_Oxy_ and P_Nox_, is thus associated with the higher contribution of the G1 sub-band to the overall emission band. This difference in the contributions of G1 and G2 sub-bands could be related to the sensitivity of 5d excited level to the host matrix depending on the glass preparation conditions. This could potentially impact the radiative relaxation channels corresponding to G1 and G2 sub-bands.

PL kinetics were taken at 450 nm under pulsed excitation at 320 nm. Whatever the conditions of sample fabrication, the obtained PL decay curves never follow a pure single exponential function. Since the Ce^3+^ doping level is low, quenching effects are excluded and such non-exponential decay can be understood as a result of simultaneous emission of many slightly non-equivalent Ce^3+^ sites in the glass matrix. Thereafter, the PL decay curves have been fitted by using a stretched exponential function. This kind of function reflects the multiple-site environment of the Ce^3+^ ions [34,35]:

(1) $$I(t)=\;y_{0}\;+\;A\;\exp\left[-{\left(\frac{t}{\tau }\right)}^{\beta }\right]$$

**Figure 4:**
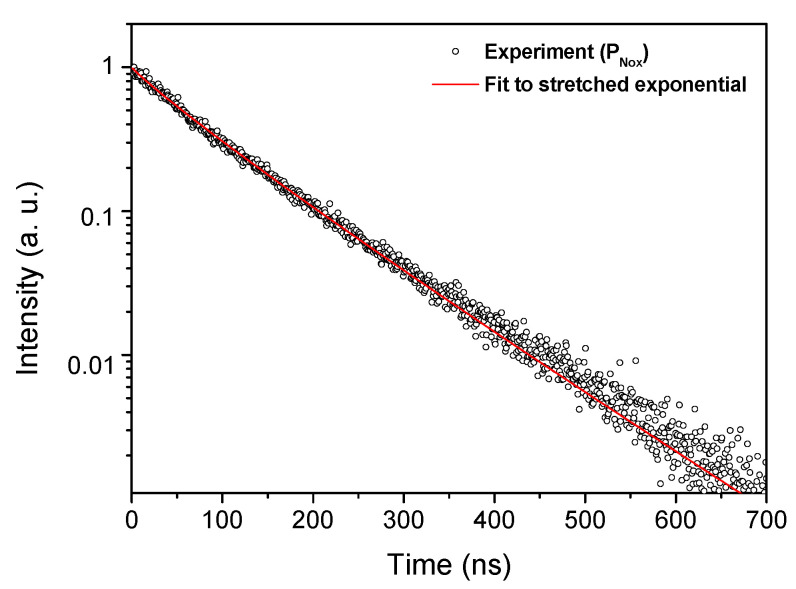
PL decay curve recorded on P_Nox_ at 450 nm wavelength, under excitation at 320 nm and its corresponding curve fitting analysis using Equation (1).

In Equation (1), *I*(*t*) states for the time evolution luminescence intensity, *y_0_* is the background, *A* designates the intensity at *t* = 0, *β* is the stretch factor and *τ* states for characteristic relaxation time. The decay curve and a fit to the function of Equation (1) are shown as an example for P_Nox_ in Figure 4. Common interpretation of such a stretched exponential behavior refers to the overall relaxation of a glass containing a number of independently relaxing species. Every species decays exponentially with one specific relaxation rate *τ* [36]. For all the Ce-doped preforms, the obtained fit parameters were summarized in Table 2. Independently of the preforms, the obtained *τ* values, in the time range of several tens of nanoseconds, are characteristic of Ce^3+^ embedded in a silica network [26,37]. This was confirmed by density functional theory (DFT) calculations, where *τ* equals 64.82 ns [38].

Absolute PL QY were determined at the excitation wavelength of 325 nm for the three Ce-doped preforms. The obtained values were 4.5 ± 0.2%, 5.8 ± 0.3% and 2.6 ± 0.1% for P_Oxy_, P_Nox_ and P_Hel_, respectively. It is noticeable that the glass fabricated under reducing conditions (P_Nox_) showed the highest PL QY. Such efficiency can be related to the higher content in Ce^3+^ ions.

All these photoluminescence investigations show that preform samples P_Oxy_, P_Hel_ and P_Nox_ contain trivalent cerium, resulting in a large emission band around 450 nm under excitation at 320 nm. The excited state lifetime remains in the range of 79–92 ns, which is characteristic for Ce^3+^ in glass samples. However, the luminescence quantum yield of these samples revealed the highest efficiency for P_Nox_ in this series. This is the reason why P_Nox_ preform was chosen as the starting point for the fabrication of fiber and X-rays sensing investigations.

### 3.2. Ce-Doped Fibers

**Figure 5:**
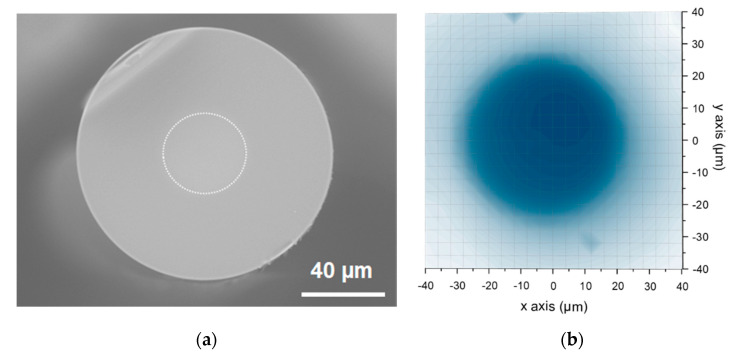
(**a**) SEM image of cross-section of the drawn fiber (the Ce-doped zone has been indicated by a white dashed circle), (**b**) Experimental mapping response of the Ce luminescence distribution along the fiber transverse cross-section: a laser excitation at 325 nm and a spatial step of 2.5 µm.

For dosimetry applications, an optical fiber was fabricated using the P_Nox_ preform with the best QY. This fiber is composed of a central non-guiding Ce-doped core surrounded by a pure silica cladding. This fiber has an outer diameter of about 125 µm while the Ce-doped core section has a diameter of about 40 µm. These considerations were verified via a microscopic characterization (SEM) of the fiber transverse section (Figure 5a) as well as by the monitoring of the PL-signal distribution of the emitting centers (Ce ions) under an excitation at 325 nm and a spatial step of 2.5 µm (Figure 5b). Indeed, the core-to-cladding refractive index difference is very small and therefore, for guiding purpose, a low refractive index polymer was applied as a coating, leading to a numerical aperture (NA) of about 0.4 for the 125 µm diameter inner cladding. The weak overlap between the guided modes and the Ce-doped zone helps to reduce the re-absorption of the light emitted by the core material.

Moreover, in the targeted dosimetry application, the signal should be emitted only in a small volume of the inner core of the fiber and should then be collected through the entire fiber length. The method of PL measurements depicted before was chosen to avoid the re-absorption of photons in the core of the fiber.

**Figure 6:**
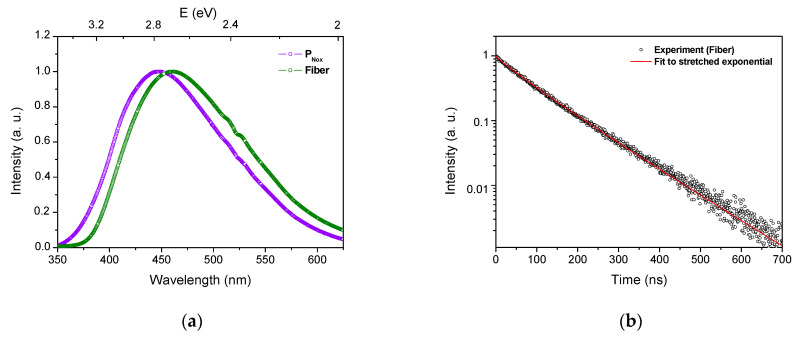
(**a**) Normalized PL spectra of Ce-doped optical fiber and its corresponding P_Nox_ preform under 320 nm laser excitation. (**b**) PL decay curve at 450 nm, after probe excitation at 320 nm and a fit using Equation (1).

PL spectra of the fiber and of its corresponding P_Nox_ preform, under 320 nm laser excitation, are reported in Figure 6a. The PL spectrum of the fiber presents a broad asymmetric band centered around 460 nm. This band shows a red shift compared to the one of P_Nox_. It can be deconvoluted into two Gaussian bands G1 and G2 peaking at 2.55 and 2.81 eV, respectively. In spite of the red shift observed between the peak positions of the fiber PL band and of its preform counterpart, the energy difference between G1 and G2 remains constant around 0.26 eV. The red shift of G1 and G2 band suggests a strengthening of the ligand field around Ce^3+^ ions after optical fiber drawing [39]. Due to the large atomic size of cerium ions in comparison with silicon atoms, it has been reported that, in silica networks, they act as network modifiers coordinating with non-bridging oxygen (NBO) [38]. Moreover, it is known that the process of fiber drawing induces the formation of NBO centers inside silica glasses [40,41]. Such an increase of the NBO concentration around Ce^3+^ ions enhances the ligand field around them and may lead to the red shift of the PL band observed for the drawn fiber sample. PL kinetics measurements were also performed on the optical fiber. Figure 6b exhibits the PL decay curve at 450 nm after excitation at 320 nm. As for the corresponding preform, this curve was fitted using the stretched exponential function, leading to *τ* and *β* values of (89.0 ± 0.4) ns and (0.926 ± 0.004), respectively. These values are close to those obtained with the corresponding P_Nox_ preform. The slight difference could be associated with the Ce^3+^ ions local environment modifications induced by the fiber drawing. Hence, PL measurements confirm the preservation of Ce^3+^ ions in the fiber core after the drawing process, with a local environment slightly differing from that of the bulk silica matrix.

**Figure 7:**
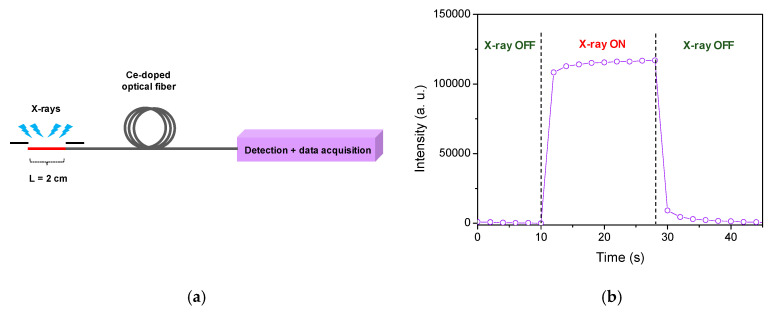
(**a**) Basic representation of the experimental setup used to evaluate the RL response of the Ce-doped optical fiber partially exposed to X-ray beam. (**b**) RL signal evolution versus time under 19.6 Gy(SiO_2_)/s dose rate.

The Ce-doped optical fiber was evaluated as an active and guiding material for remote X-ray dosimetry in all-fiber configuration. A four-meter-long Ce-doped fiber was applied for RL measurements, where only a 2 cm-long piece was put in a calibrated position of the X-ray irradiator (Figure 7a). The entire rest of the fiber guided the RL signal generated by X-rays towards a single photon avalanche detector associated with a rapid counting module. At the Figure 7b, we illustrated the time evolution of the RL signal at a dose rate of 19.6 Gy(SiO_2_)/s. As the X irradiation begins, the RL signal increases and it takes 2 s to reach about 95% of its maximum. After switching off the X-ray, the signal is going down to zero in approximately 20 s.

**Figure 8:**
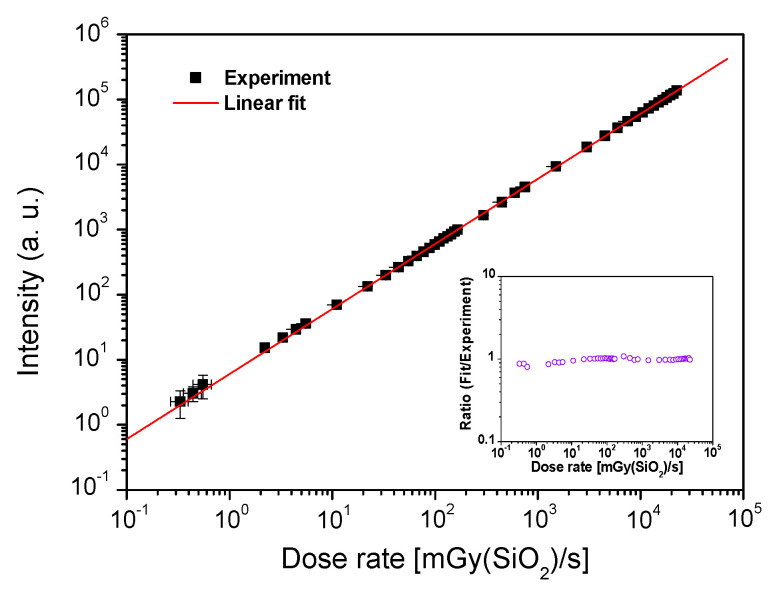
Averaged RL signal of the Ce^3+^-doped optical fiber against the X-ray dose rate. In the inset, a plot of the ratio between fit and experimental intensity values as a function of the dose rate.

In Figure 8, the averaged values of the intensity calculated in the region of the plateau are reported as a function of the X-ray dose rate. The linearity of the RL signal intensity *versus* the dose rate is depicted in the range from 330 µGy(SiO_2_)/s to at least 22.6 Gy(SiO_2_)/s. This maximum limit is the highest dose rate attainable with the experimental setup in LABHX facility (with respect to our experimental conditions: dose rate homogeneity over the entire sample spatial zone). We cannot exclude that the signal linearity is extended over this limit. The goodness of fit was checked by R^2^ value (0.99955). The plot of the ratio between fitted and experimental values was reported as a function of the dose rate in the inset of Figure 8. The small variations of experimental to fitted results reflect the linearity confidence. The measurement uncertainty was ≈10%, essentially due to the accuracy of the ionization chamber used to calibrate the dose rate. Otherwise the reproducibility of measurements was checked for several dose rate values. To the best of our knowledge, such a linear response is reported for the first time over five decades, with a Ce^3+^-doped silica optical fiber in an all-fibred dosimetry configuration.

**Figure 9:**
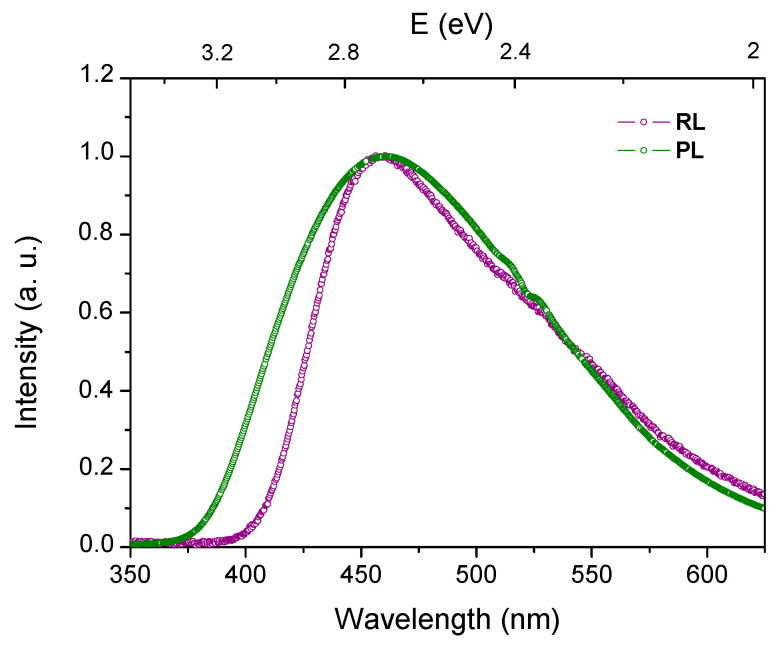
Ce-doped optical fiber responses: normalized typical RL spectrum and the PL one obtained under 320 nm excitation.

The RL signal origin was checked through the RL spectrum recorded by connecting the fiber to the spectrometer previously used for PL measurements. Figure 9 shows a typical RL spectrum of Ce-doped fiber, which is compared to the PL spectrum, obtained under 320 nm excitation. Both RL and PL signals exhibit a maximum around 460 nm, representative of Ce^3+^ ions emission. However, the RL band shows a reduced full width at half maximum (FWHM), especially in the higher-energy part of the spectrum. The FWHM change could be attributed to various excitation/emission channels involved in the PL and RL processes. In the PL mechanism, the incident photons selectively excite the trivalent cerium ions, while in the RL process, X-rays initially generate electron-hole pairs, which then relax through defect trapping levels before the final radiative recombination on the excited Ce^3+^ centers. Besides, we cannot exclude re-absorption effect in the case of RL spectrum. Indeed, the RL signal was guided through a longer fiber length compared to length used for PL measurement. This could modify the RL band shape. Anyway, from the global profiles of RL and PL spectra, we can consider that for both phenomena, the final transition occurs from the lowest 5d excited state to 4f ground states ^2^F_5/2_ and ^2^F_7/2_ of trivalent Ce^3+^ ions.

## 4. Conclusions

In this study, cerium-doped silica preforms have been prepared using MCVD in combination with a solution doping approach. Depending on the fabrication atmosphere, three kinds of preforms were obtained. Raman investigations have not shown significant structural variations between these preforms. The sample fabricated under reducing atmosphere showed the highest Ce concentration, mainly present in the cerous form (Ce^3+^) and leading to a higher PL quantum yield. An optical fiber was fabricated from this sample and the Ce^3+^ presence (in the fiber core) was validated via PL measurements. The optical activity of this fiber was then exploited for X-ray radiation dosimetry in a remote all-fibred configuration. The RL signal displays a linear behavior versus the dose rate, at least in the [330 µGy(SiO_2_)/s–22.6 Gy(SiO_2_)/s] range, namely covering five decades. These results reveal the potential of such a fiber, used as both active and guiding material, for real-time remote ionizing radiation dosimetry. Moreover, this kind of sensor/dosimeter can be used in high spatial resolution applications thanks to the small sensitive volumes achieved with optical fibers.

## Figures and Tables

**Table 1:** Normalized areas of Gaussian sub-bands (in %) composing the PL spectra of the three Ce-doped preforms under excitation at 320 nm.

Gaussian Band	P_Oxy_	P_Nox_	P_Hel_
G1 (2.64 eV)	67.85	68.59	85.51
G2 (2.9 eV)	32.15	31.41	14.49

**Table 2:** Parameters obtained from decay curve fitting using Equation (1).

Sample	*τ* [ns]	*β*
P_Oxy_	79.6 ± 0.5	0.904 ± 0.005
P_Nox_	83.3 ± 0.5	0.920 ± 0.006
P_Hel_	92.3 ± 0.6	0.945 ± 0.006

## Data Availability

The data supporting the findings of this study are available from the corresponding authors upon reasonable request.
